# On Repeated Doses of Castor Oil, Especially in Certain Skin Diseases in Children*Read in the Section on Diseases of Children at the Thirty-sixth Annual Meeting of the American Medical Association.

**Published:** 1885-09-20

**Authors:** L. Duncan Bulcley

**Affiliations:** New York


### ON REPEATED DOSES OF CASTOR OIL, ESPECIALLY IN CERTAIN SKIN DISEASES IN CHILDREN.*

By L. DUNCAN BULCLEY, A. M., M. D., OF NEW YORK.

Castor oil is such a common and well known remedy that it would seem that but little could be written with regard to its employment in medicine. Every one is thoroughly acquainted with its characters, dosage, and use, and even the laity appreciate its value and employ it freely without medical advice. But, as far as I

*Read in the Section on Diseases of Children at the Thirty-sixth Annual Meeting of the American Medical Association.

can learn, its use is confined to the administration of single doses, now and again, as necessity demands to remove offending contents of the gastro-intestinal tract, with the single exception, perhaps, of the well known mode of its employment in emulsion, in frequent doses, for the relief of diarrhœa and dysentery.

In the present communication, however, I wish to call attention to the value of castor oil in certain cases, in which frequent and repeated doses of some size, and continued even for a considerable period, will produce effects difficult to obtain by other means. I will first give in brief the notes of a case—that of a child—in whom its virtues were strikingly exhibited, and which especially confirmed me in the practice now advocated :

The child, aged six years and a half, was brought to me suffering from chronic uticaria, which had existed, off and on, for some little time, and had resisted good treatment at the hands of others. The child was fairly developed, but of an earthy complexion ; was restless, peevish, and complained continually of great fatigue. She had previously taken as a tonic an iron mixture, and also a mixture of rhubarb and soda, but without benefit to her health ; she had recently been in the South, and had contracted malaria, which quinine held in check with difficulty. She was found to be the subject of urticaria, the wheals developing rapidly at any time of day, and from no especial cause. When seen there was not very much of urticarial element present, but there was more or less of the papillary eruption left after the disappearance of the former lesions, constituting the disease known as urticaria papulosa. Her pulse stood at 94, of fair strength, the tongue was moderately clean. For some time previously she had complained of pain in the right abdominal region, and would call attention to it frequently. On percussion of the abdomen the right iliac region was found to present very considerable dulness, extending well up to the liver, and somewhat into the transverse colon ; there was no special dulness on the left side. She had complained of diarrhœa, the movements, however, were ineffective, and often more or less lumpy.

She was ordered to take a teaspoonful of castor oil every night, and was also given an emulsion of castor oil with gum arabic and sugar, and a little opium to check excessive action. Twelve days later she was brought to the office, having taken a teaspoonful of castor oil every night in the intervening time. This had never acted more than three times daily, and generally not more than once or twice. The diarrhœa had been checked in two days, but she still complained of pain in the abdomen, sometimes in the left side, with which she had two attacks of crying. She looked very much better in general appearance, with less of an earthy color, and her appetite, which before had failed entirely, was now good. On percussion the abdominal dulness was decidedly less, but still marked ; the stools had contained more or less of lumps with each movement. The administration of the oil every night was continued, and two grains of quinine were ordered to be taken before each meal. About a week later she was again seen, and appeared to be still better in health ; the pulse was 80, and the tongue clean. The oil had been increased to a teaspoonful and a half each night, and lumps had ceased to appear in the stools, which presented only healthy fæcal matter. The pain had ceased entirely in the abdomen, but there was now more general dulness over the entire abdominal region. It was then attempted to administer again to her an iron mixture, with the continuance of the oil, but this shortly disagreed, causing her to sleep badly and to be restless and irritable, and it was discontinued, and an alkaline and bitter tonic given in its place. About one month after the commencement of the castor oil it was recorded that she looked remarkably well, fat and round in the face ; had lost the pale, earthy look, and had gained two pounds and a half in flesh ; the abdomen was resonant all over.

The castor oil was still continued in moderate doses, producing one or two quite healthy movements. Shortly after this the oil was omitted for a few nights, contrary to directions, and she began to be again troubled in her sleep, and felt poorly, and the urticaria, which had disappeared very shortly after beginning the oil, returned in considerable degree. The abdomen was found to be again dull on the right side, and the castor oil was returned to, with the result of producing shortly a resonant abdomen. She was then given a mixture containing lactopeptine after each meal, which had the effect of inducing regular healthy action of the bowels. The urticaria had ceased with the last administration of castor oil, and the child remained afterward in m[illegible]h better health ; but on another occasion somewhat later the [illegible]dominal indigestion returned, and the castor oil was again retu[illegible]d to with good effects.

In this case the little patient took the castor oil continuously for over one month, and for shorter periods on three other occasions. In a number of cases of infantile eczema, even where there was not the evidence of obstructed bowel excretion, I have administered castor oil, as in the preceding case, with great advantage, in doses varying according to the age of the patient and the necessities of the case, never, however, so as to produce purgation, but only to excite a somewhat increased and healthy discharge from the intestinal canal.

In one case—that of a boy aged about eight years—the impetiginous eczema, which had cropped out from time to time upon the face and scalp, yielded most happily to this course of treatment ; but, as happens so frequently in public practice, the neglect of treatment for a period, together with bad dietary and hygienic surroundings, necessitated its use on several occasions. The child had also ulceration of the cornea, and at several visits the mother would remark that this ulceration, which had resisted local treatment at the hands of oculists, yielded in a remarkable manner each time when the oil was administered.

Acting on the suggestion mentioned in the last case, I employed this treatment by repeated doses of castor oil with very great advantage in a case of acne rosacea, complicated with persistent and rebellious corneal ulcerations, in brief, as follows :

Mrs. R——, aged twenty-six, had had acne simplex and rosacea for several years, connected with and dependent upon great constipation, uterine disorder, and much general debility. For many years she had been troubled with the eyes, having recurring ulceration of the cornea, and much conjunctivitis very frequently. For this she had been under skilled treatment, but never with anything more than a temporary improvement. Time and again during the treatment of her acne rosacea she came with the eyes almost useless, and suffering greatly from them. This condition was found to vary considerably with the state of her digestive organs and with the eruption upon her face, and several times the eyes ceased to give trouble for quite a period when the other symptoms improved. Soon after the occurrence of the case just mentioned, she had another severe attack of ulceration of the cornea and conjunctival irritation. She was then placed upon castor oil, taken every night in doses of from two to four teaspoonfuls. This was taken continuously for something over a month, and the record was made that it helped her general condition greatly, and her eyes in particular. The eruption upon her face was better than it had been for a very long time. Castor oil was still continued in connection with tonics, which before had proved inefficacious alone, and nearly a month subsequently it was noted that her general health was better than it had been for a long time, and that she had as yet had no return of the difficulty with the eyes. She was seen subsequently, after an interval of about a year, and stated that from time to time she had returned to the castor oil treatment immediately that the eyes gave any indication of inflammation, and always with the happiest effect; a few doses sufficed to check this trouble, which before had invariably run on to producing very serious inconvenience and distress.

Castor oil is also often of value in other conditions not associated with disease of the skin, as in the following rather striking case of chronic and recurrent tonsillitis:

L. B——, a very bright, rather precocious, child of seven years, came under my care December 14, 1883. He began to have attacks of throat trouble when but one year old, the tonsils becoming swollen and covered with small yellow points, as in ordinary follicular tonsillitis. The attacks lasted one week, during which time he was generally in bed most of the time, and from that period until the date of his visit, at seven years of age, he had had recurrences of this throat inflammation every month, except when away from home in the summer, with very few exceptions, occasionally missing a month or two under active treatment. With these attacks he would always have pain in the stomach, and nausea, and, latterly, very great pain in the head. The attacks were often preceded by slight chills, and before their occurrence the movements from the bowels would become lighter in color, and clay-like, up to the time of the attack. Between the attacks the bowels were apt to be constipated, the sleep bad and restless. During the attacks the urine would become very high colored, and afterward would throw down abundant deposits. The last attack had begun two weeks before his visit, he having been out of bed only six days when brought for treatment from his home in New York. When first seen, the tonsils were found to be enlarged and covered with small cicatrices, and presented a few very small yellow points. On percussion over the abdomen the liver was found to be considerably enlarged, protruding plainly below the line of the ribs. The spleen was two or three times its natural size, and the abdomen appeared full and rather tense with gas, but considerable dulness was discovered on deep percussion. In several places there was much tenderness on deep pressure.

He was directed to take two teaspoonfuls of castor oil every night, and no other treatment. Three days later it was recorded that the castor oil had agreed perfectly, and had acted gently upon the bowels, producing about one movement daily, which was a little loose, the first movements which occurred being lumpy. He then returned to his home in a somewhat malarious district, continuing the oil, as before, every night, but in somewhat increased doses. One month from the beginning of the previous attack, it was thought that he had a slight chill, but he did not take to bed as before. The next day there was some little fever in the afternoon, and nausea at night, with a little soreness of the throat, which, however, passed off the next day. He had omitted a dose or two of the oil just previous to this slight attack. The movements had been somewhat light colored, but better than previously. He was brought to the city from some 200 miles distance, the second day after the slight chill just mentioned—that is, during the period when he should have had his attack. The throat was then seen to be but little red ; there was no ulceration, and he complained of no soreness. He was then given a rhubarb and soda mixture in place of the castor oil, but this failed to be as efficacious ; and two weeks after the last note his throat again became a little sore, although there was nothing to be seen. The movements were again very light colored. The castor oil was then returned to at night, and a mixture of chlorate of potash and iron was given for the immediate relief of the threatened sore throat. The next day or two he had a little fever, but was able to come to the office, and shortly after he went to a country place quite free from malaria, but did not stay longer than a week or so. He then returned to his home, continued the castor oil every night, and taking also the syrup of the lactophosphate of lime after each meal. On March 22 it was recorded that he had taken the castor oil continuously, two teaspoonfuls each night, and that he had not had any further difficulty with the throat, it being now fully eight weeks since there had been any trouble at all. The movements were still inclined to be rather light colored, but, although living at home, he remained entirely free from his throat disease until June 6th. He then had a slight attack of fever with a little headache and sore throat, which, however, did not confine him to bed, there being no yellow points on the tonsils as before. He had become rather careless in diet, and it was learned that the location and house in which he lived were in a bad sanitary condition.

Other cases could be cited in children up to the age of ten years in which the castor oil was administered upon this plan for periods varying from two to four weeks or more, with the results of removing abdominal dulness, and with it debility and a train of unpleasant symptoms. One particular case may be alluded to, in which a little child, three years and a half old, who had eczema in infancy and urticaria subsequently, was very greatly improved in health by the administration of the oil for a period of over five weeks. During this time her appetite, which had failed entirely, returned with vigor. She gained in flesh and color, and at the end of the period mentioned her condition was as different from that at first as could be imagined, greater than one often sees produced by any course of medication. Various tonics previously given had failed to be of much benefit.

The cases which I have here cited, together with a number of others, show that castor oil may be taken with advantage repeatedly for a considerable period of time. In the boy with the sore throat, this treatment was carried out for a period of almost six months, with few intermissions, he repeatedly taking it for a month or more without intermission.

In regard to the mode of action of the oil; in these cases it acts unquestionably as a stimulant to the abdominal organs, the color and character of the movements from the bowels being the indication with regard to its use. In those cases, which I believe to be not infrequent, where there is intestinal torpor followed by greater or less accumulation of fæces in the large intestine at either side or throughout its entire length, this plan of treatment is peculiarly happy and beneficial in its effects. The apparently tonic effects following this course of treatment must be attributed largely to the improved absorption and assimilation which take place in the gastro-intestinal tract, although it is quite possible that some portion of the oil thus taken acts as a direct nutrient. Many cases of skin disease, especially urticaria, are undoubtedly attributable to reflex irritation, having its origin in the intestinal canal, and undoubtedly many cases of tonsillitis have their origin in like manner. In repeated instances in my own family have I observed that beginning tonsillitis could be broken up almost immediately by the free administration of castor oil.

With regard to the administration of this remedy, which is so often considered nauseous and repulsive, a few words may be added. Specimens of castor oil undoubtedly differ not a little in their qualities, and care should be exercised to secure a sweet, pure and fresh quality of cold-pressed castor oil. To adults this drug is undoubtedly repulsive, but in view of the value of the gain which often results from its administration, I have found little difficulty in having a number of adults take it as here described, for a greater or shorter length of time. To children, however, if especial attention is not called to it, the remedy is not as repulsive as to those of older years, and young children are often seen to be very fond of it. In several of my cases, in which it was continued for a considerable period of time, the children remarked that they did not find it at all an unpleasant dose to take.

With regard to the mode of administering castor oil, there are very many plans which may be adopted. I may first remark, however, that it is important that the remedy should be taken alone, and not with any other substance which may interfere with its action. I object most decidedly to having spirit, wine, or any such substances added to it, and prefer even that it should not be given in conjunction with orange, milk, coffee, etc. The plan I have usually adopted is as follows: The patient takes a sip of very cold ice-water or a small lump of ice, holding it in the mouth for a moment, and the oil is immediately taken in a single dose from a large spoon. The lips are then quickly and very firmly wiped or rubbed with a towel, and a drink of ice-water is taken instantly afterwards. In this way those who have shuddered at the idea of taking the oil, have found that it gave them little annoyance, and in this manner it is left free to act in the stomach without the presence of substances which might interfere with its operation.

In conclusion, I would add that in presenting this subject of the repeated administration of castor oil, I do not desire to place too much emphasis upon its value, nor to recommend it as a panacea even for the conditions previously alluded to. In almost all the cases in which I have employed it, other remedies have been given either conjointly or from time to time alternated with it, and to them, undoubtedly, must often be ascribed some measure of the good result obtained. On the other hand, I can affirm that, for certain conditions—such as may be learned from the cases cited—I have found no remedy to equal it, and I believe that in these and in many other conditions the prolonged use of repeated doses of castor oil will be found to serve a valuable purpose in our efforts to combat disease.—*Jour. American Med. Association.*

